# Human rights in patient care: drug treatment and punishment in Russia

**DOI:** 10.1186/s40985-018-0088-5

**Published:** 2018-06-01

**Authors:** Mikhail Golichenko, Sandra Ka Hon Chu

**Affiliations:** Canadian HIV/AIDS Legal Network, Toronto, Canada

**Keywords:** Russia, Drug treatment, Human rights in patient care

## Abstract

An inherent feature of drug control in many countries has been an excessive emphasis on punitive measures at the expense of public health. At its most extreme, this approach has reduced health services for people who use drugs to an extension of the drug control system. In these environments, health services are punitive rather than supportive for people who use drugs, especially those who are drug dependent.

In Russia, the government’s official policy towards drug use is one of “social intolerance,” which seeks to legitimize and encourage societal ill treatment of people who use drugs. In practice, this policy has materialized as widespread and systematic human rights violations of people who use drugs, including by subjecting them to unscientific and ideologically driven methods of drug prevention and treatment and denying them access to essential medicines and services. While such human rights violations are well-documented, there have been no attempts to date to consider the consequences of this approach through the lens of human rights in patient care. This concept brings together the rights of both patients and providers and interrogates the role of the state on the relationship between two core groups: drug-dependent people and drug treatment doctors or “narcologists” in Russia.

In this article, we apply the concept of human rights in patient care to consider the narcologist’s role in punitive drug policy and human rights violations against people who use drugs and to analyze how punitive drug policy manifests as human rights violations against narcologists themselves, who lose their professional independence and their ability to work according to professional standards and ethical norms. We conclude that both people who use drugs and narcologists suffer from punitive drug policy and should unite their efforts to ensure drug policy does not undermine patients’ health and human rights.

## Background

An inherent feature of drug control in many countries has been an excessive emphasis on punitive measures at the expense of public health. At its most extreme, this approach has reduced health services for people who use drugs (PWUD) to an extension of the drug control system, where health services are punitive rather than supportive for PWUD.

In Russia, the seminal international drug control document—the *Single Convention on Narcotic Drugs, 1961*, which states in its Preamble that “addiction to narcotic drugs constitutes a serious evil for the individual and is fraught with social and economic danger to mankind”—sets the principal tone of the Russian drug control system [1]. The government’s official policy towards drug use is one of “social intolerance” [2], which seeks to legitimize and encourage societal ill treatment of PWUD. Research suggests that the Russian public supports this policy [3]. Non-sterile drug injection remains the leading cause of HIV infection and nearly one quarter (23%) of adult prisoners were convicted for drug-related offenses [4]. Russian and international civil society organizations have documented such human rights violations and published reports describing the grave impacts of Russian drug treatment and care on the human rights of PWUD, including widespread and systematic torture and ill treatment, and the denial of access to essential medicines and services [5–9]. The United Nations Human Rights Treaty Bodies and the European Court of Human Rights have also recognized these human rights violations. [5] However, there have been no attempts to date to consider the consequences of this approach on the relationship between two core groups: drug-dependent people (DDP) and drug treatment doctors or “narcologists” in Russia.

In this article, we apply the concept of human rights in patient care to analyze narcologists’ role in punitive drug policy and human rights violations against PWUD. We begin by providing an overview of the professional regulations and code of ethics governing Russian narcologists and their potential to address the extreme vulnerability of PWUD—and especially DDP—to human rights violations. While this regulatory framework encourages narcologists to fulfill their legal and ethical responsibility to ensure that their patients are treated according to human rights standards, in practice, this rarely happens. We attribute this to the phenomena of distorted “dual loyalty” of narcologists and describe the legal and policy drivers underpinning this distortion. We further analyze how punitive drug policy in Russia manifests as human rights violations against narcologists themselves and conclude that both PWUD and narcologists suffer from punitive drug policy and should unite their efforts to ensure drug policy does not undermine patients’ health and human rights.

### The concept of human rights in patient care

“Human rights in patient care” refers to the application of human rights principles to the context of patient care. Recognition of the interrelated and interconnected nature of the human rights of patients and health-care providers is a cornerstone of this concept, which also focuses on the role of the state to respect, protect, and fulfill the human rights of patients and health-care providers. Apart from placing more emphasis on patients’ agency and autonomy, the concept of human rights in patient care focuses on the most marginalized and vulnerable patients, such as PWUD, including those who are drug dependent [10].

Human rights in patient care benefit both patients and health-care providers by recognizing particular rights. For patients, this includes the rights to liberty and security of person (article 9(1) of the *International Covenant on Civil and Political Rights* (ICCPR) [11] and article 5(1) of the *European Convention on Human Rights* (ECHR)) [12], to information (article 19(2), ICCPR and article 10(1), ECHR), to be free from ill treatment (article 7, ICCPR and article 3, ECHR); to life (article 6(1), ICCPR and article 2, ECHR), to health (article 12 of the *International Covenant on Economic, Social and Cultural Rights* (ICESCR) [13] and article 12 of the *Convention on the Elimination of All Forms of Discrimination against Women* (CEDAW)) [14], and to non-discrimination (article 21(1), ICCPR, article 2(2), ICESCR and article 14, ECHR). For health-care providers, this includes the rights to freedom of association (article 22, ICCPR and article 11, ECHR), to the enjoyment of decent work conditions (article 7, ICESCR), and to due process (article 14(1), ICCPR and article 6(1), ECHR) [15].

Notably, this concept lays bare the competing interests between punitive drug policy and public health, which result in situations where narcologists have concurrent—and often mutually exclusive—obligations to law enforcement on one hand and to their patients on the other. In health services targeting PWUD, health-care providers must often balance state objectives such as drugs and crime prevention with their clients’ rights and freedoms. A health-care provider’s “simultaneous obligations, express or implied, to a patient and to a third party, often the state,” is called “dual loyalty” [10]. In Russia, such dual loyalty severely exacerbates drug-related harms to individuals and to narcologists, who lose their professional independence and their ability to work according to professional standards and ethical norms. This is a major factor contributing to human rights violations against PWUD, as well as to violations of narcologists’ human rights—an outcome that has ripple effects beyond Russia [16].

## Case presentation

### Professional regulations and code of ethics for narcologists in Russia

Since the 1970s, Russian narcologists have formed a separate subset of medical professionals in psychiatry [17]. They are regulated by the same rules of professional ethics as psychiatrists, which require narcologists to respect all human rights of their patients, including those listed above (The referenced laws define narcologists’ obligations to respect and fulfill human rights of patients [18–21]). Russian narcologists have an ethical responsibility to develop relationships with their clients based on partnership, mutual trust, and responsibilities. They also have a right and a professional responsibility to maintain their professional independence and to act according to the best interests of their patients, and rights to form professional associations and to facilitate the development of their profession and science. Narcologists in the Russian Federation are thus empowered by legal and ethical instruments to carry out their professional activities according to a framework of human rights in patient care, whereby the rights of patients are complemented by the rights and responsibilities of doctors to form therapeutic partnerships with their patients to achieve the most desirable treatment outcome.

The unique vulnerability of PWUD to human rights violations and punitive drug laws and drug enforcement, however, affects the practical realization of Russian narcologists’ professional rights and responsibilities [5]. While the vulnerability of PWUD to human rights violations should impose a special legal and moral obligation on narcologists to respect and protect the human rights of their patients, Russia’s drug laws and drug enforcement strongly influence the methods and the environment in which drug dependence treatment is rendered, to the extent that drug dependence treatment in Russia does not conform to a framework of human rights in patient care.

### Narcologists and punitive drug laws and drug enforcement

Most leaders of Russian narcology are well known for their robust opposition to evidence-based approaches to drug dependence, including opioid substitution therapy (OST), recommended by the World Health Organization (WHO) as one of the most effective forms of opioid dependence treatment, and proven HIV prevention interventions such as harm reduction programs [22]. Eduard Babayan and Nikolay Ivanets, for example, are two senior narcologists who played a prominent role in establishing and maintaining the current legal ban against OST in Russia and the country’s broader punitive approach to drug treatment [23].

Significant legal and policy restrictions on Russian narcologists to practice science-based drug treatment and to participate in scientific debates have also isolated Russian narcologists from scientific developments on drug dependence treatment, and led to the development of radically different approaches to such treatment [24]. Isolated from international science and peer discourse, and prohibited from access to evidence-based methods of drug dependence treatment, Russian narcologists have developed and patented unproven, life-threatening methods of drug dependence treatment, such as electroshock therapy and comatose therapy and heating up a patient’s body to 43 °C—described by one academic as “science-decorated shamanism” [25]. Because such brutal practices conform with Russia’s punitive approach to drug policy, they have been largely immune to scientific scrutiny.

Recently, this opposition to science and human rights reached a new frontier. In 2010, Russia’s Chief Narcologist announced his endeavor to create a four-level system of “social pressure” in order to respond to the country’s “drug problem” [26]. The first level of this system involves “early detection” of drug use by way of school and workplace testing; the second level is voluntary drug treatment; the third level is compulsory treatment by referral from the criminal justice system; and the fourth level is compulsory treatment within the criminal justice system. By 2013, this system was fully implemented as state policy. Despite the fact that compulsory drug treatment was proclaimed unconstitutional in Russia in 1989, the punitive principles underlying Russia’s current drug policy allowed for widespread ignorance of this fact—not an unusual practice in Russia [27]. Correspondingly, in 2013–2014, several federal laws and regulations were amended to establish compulsory drug treatment [28–30], purportedly to motivate DDP and people who use illegal drugs to undergo medical treatment and rehabilitation [31]. For example, these amendments empower law enforcement agencies to coerce PWUD to undergo drug treatment and rehabilitation, empower courts to issue drug treatment orders to people who commit drug-related administrative offenses (such as non-medical use of narcotic drugs or possession of insignificant amounts of narcotic drugs) or to DDP who commit minor crimes (such as theft or the possession of significant amounts of drugs for personal use), introduce administrative punishment of up to 30 days of imprisonment for evasion of court-imposed drug treatment or rehabilitation, and require drug treatment and rehabilitation organizations to report to police those patients who do not fulfill court-imposed treatment or rehabilitation orders.

Analysis of court statistics demonstrates that the 2013–2014 amendments have not led to the expected outcome of “motivating” PWUD to undergo drug treatment or rehabilitation. Only about 2% of people convicted for drug administrative offenses chose to undergo treatment rather than punishment (about 1500 out of more than 70,000) [32] and only about 1% of 48,557 people who were involuntarily ordered to undergo drug dependence treatment remained drug-free within a year or more after treatment. Publically available judgments indicate that people have either simply not shown up for their appointments with narcologists or failed to visit narcologists after diagnostics (after which narcologists report truant patients to the police) [33]. Despite this obvious ineffectiveness, narcologists continue to express strong support for this system of “social pressure.” In June 2017, the Ministry of Health of the Russian Federation sponsored a large conference of narcologists. The conference’s final resolution included recommendations to health institutions in Russia to form a system of social pressure for people who use psychoactive substances, including a mechanism of legal “motivation” for treatment and rehabilitation as an alternative to administrative and criminal liability for people committing drug crimes. The same conference endorsed a bill to be introduced to the Federal Parliament in order to expand the coercive treatment measures of 2013–2014 to “problem alcohol users” [34].

The absence of evidence-based drug dependence treatment in Russia has rendered treatment an ineffective and an unattractive option for most patients. Medical statistics reveal the declining number of patients seeking medical treatment with state and municipal drug treatment clinics, at a time when there is an increasing number of people who use or depend on drugs [35]. Because of this, doctors have little choice but to resort to the use of coercion in order to force—and retain—patients in treatment. By exploiting punitive drug policy and drug treatment approaches, narcologists are able to ensure the inflow and retention of patients.

Russian narcologists’ desire to toughen already punitive approaches to drug use and drug dependence goes far beyond the concept of dual loyalty discussed earlier. Rather, the majority of Russia’s narcologists have voluntarily or under pressure stripped themselves of their professional independence and effectively extinguished any notion of human rights in patient care. Dual loyalty is distorted to such a degree that doctors’ allegiance to the state objective of a “drug-free world” nullifies their legal and professional obligations to their patients. The following legal and policy drivers explain this dilemma in further detail.

i. Drug dependence treatment is legally subordinated to law enforcement and regulated by the law concerning drug control and enforcement [36]. This law defines drug dependence treatment and drug dependence rehabilitation, establishes the ban on OST, and authorizes law enforcement agencies to register and regulate patients. At the same time, the health system is legally subordinate to the Minister of the Interior within the State Anti-Drug Committee, which was formed in 2007 as an umbrella coordinating body for the drug-related work of 31 ministries, as well as subsidiary Territorial Anti-Drug Commissions in every region of Russia. Thus, all decisions of any relevance to drug control matters by any branch of government are under the control of the Minister of the Interior [37].
ii. Drug dependence is positioned as both a health condition and an offense, thus warranting exceptional control and coercion. According to Eduard Babayan, one of the founding fathers of the current system of drug dependence treatment in Russia, “Those suffering from drug and alcohol addiction violate societal moral standards on purpose, voluntar[ily] bringing themselves to the state of sickness. That’s why society’s actions towards these people cannot be the same as actions on medical assistance to other categories of patients” [38]. By positioning drug dependence as both a health condition and an offense, narcologists can justify the ineffectiveness of their care and their inaction in improving drug dependence treatment, including their failure to advocate to lift the legal ban on drug dependence treatment methods such as OST. As current Chief Drug Treatment Doctor Evgeny Bruin has indicated, drug dependence is a particular form of delusion when patients are unable to understand what they are doing; coercion (in the form of mandatory drug dependence treatment) is thus a reasonable measure to save patients from themselves [39].

In particular, maintaining narcologists’ control over their patients was the main purpose of the drug user registry—a data-filing system carrying the personal data of all drug treatment patients, for whom certain rights are automatically restricted [40]. The registry operated pursuant to a 1998 USSR Ministry of Health Order until it was amended by a 2015 Order of the Russian Ministry of Health stipulating that no patient should enter the drug registry without his/her voluntary, informed consent [41]. However, as recent court case files suggest, narcologists ignore this requirement and continue operating the registry as before [42].^1^ PWUD can be registered based on letters from the police to a narcologist [43]. In some cases, the only “help” a patient has received from a narcologist has been registration, followed by restrictions of their rights [44]. For instance, inclusion on the drug user registry could be sufficient grounds to deprive or limit a person’s parental rights [45]. This has had an especially profound negative effect on prеgnant women who use drugs, who avoid contact with the health-care system for fear of losing their parental rights, including child custody. In some regions, prosecutors request medical data from narcologists and child protection bodies to carry out this very deprivation [46]. In many cases, police have used medical data from the registry to arrest PWUD [47]. Yet, narcologists continue to operate the drug user registry despite the well-documented fact that it has discouraged PWUD from seeking help from the public health system.

In very rare cases, narcologists have chosen to fight for the rights of their patients when medical data is used by law enforcement [48, 49]. More often, however, narcologists and health officials argue that DDP present an imminent, serious threat to the public, factors that in their opinion justify the disclosure of medical information to law enforcement for permanent supervision. Such arguments were brought against patients who complained that the disclosure of medical information to law enforcement would lead to the erosion of trust with their narcologists [50, 51].

iii. The legal obligation of patients to “preserve their health” (which might include an obligation to comply with a doctor’s orders or to take prescribed steps to protect their health) permits doctors to blame patients for not fulfilling this obligation. This Soviet-era obligation was re-introduced into law in November 2011[18, 52, 53].^2^ Armed with this legal obligation, doctors have shifted the focus of public discourse from the availability, accessibility, and quality of health services to patients’ behavior that is deemed to be morally reprehensible and can lead to certain health conditions, such as drug dependence or HIV. Following this logic, doctors can expel drug-dependent patients with HIV and tuberculosis from clinics for violation of clinic rules when their patients continue using drugs because no effective drug dependence treatment is available [54].

Depending on the circumstances of a particular case, and especially in cases involving DDP, the legal obligation to “preserve one’s health” can lead to violations of the rights to liberty and security of a person (e.g., in cases of compulsory treatment and drug detention centers), to health (e.g., when patients are precluded from evidence-based health services), to remedy and to due process (e.g., when there is no recourse to challenge the lack of access to services appropriate for chronic health conditions), and to non-discrimination (e.g., when a chronic health condition is an obstacle to health care, as in cases of patients who are expelled from tuberculosis clinics for using drugs). In some cases, this may even lead to violations of the rights to freedom from ill treatment and to life. For example, lack of access to OST and medical practitioners’ desire to coerce a patient into abstinence may put a patient’s life at serious risk. One such case involved a drug-dependent woman who was left without medical help because narcologists and gynecologists had no access to evidence-based methods of drug dependence treatment for pregnant women who use drugs. The legal system governing drug use offered the patient only the emaciated options of either terminating her pregnancy or immediate abstinence, despite the fact that such abstinence carried significant risks for the fetus. The case demonstrates how the undue allegiance of medical practitioners to state-promoted abstinence-based drug treatment blinded doctors to the specific needs of a pregnant woman, whose life, as a result, was at immense risk [55].

### Nobody wins: the impact on the rights of patients and narcologists

As noted above, human rights organizations and UN bodies have documented human rights violations against PWUD in Russia, including the absence of drug dependence treatment for people living with HIV and tuberculosis [56], the use of unscientific methods and the drug user registry in drug dependence treatment [57], and the prohibition on OST [57, 58]. Moreover, the UN Committee on Economic, Social and Cultural Rights (CESCR) has urged Russia to apply a human rights-based approach to PWUD so that they do not forfeit their right to health [59, 60], while the UN Human Rights Committee has recommended that Russia provide effective drug dependence treatment to people in police custody [61] and the UN Committee on the Elimination of Discrimination against Women has recommended that Russia provide drug-dependent women access to OST [62]. As of September 2017, there were also at least five applications pending before the European Court of Human Rights concerning the human rights of PWUD.^3^

However, human rights violations arising from punitive drug policy are not limited to PWUD. Arguably, narcologists’ human rights are also infringed when Russian drug laws criminally prohibit evidence-based drug dependence treatment such as OST, thus subjecting narcologists who are willing to provide OST to their patients to life imprisonment for drug trafficking. Narcologists are also prohibited from openly supporting harm reduction activities, such as needle and syringe programs, because such support can lead to administrative or criminal sanctions for violations of drug propaganda laws [63, 64]. According to a former Chief Narcologist, Nikolay Ivanets, Russian narcologists would never speak in favor of OST because of the risks of prosecution [65]. Russian narcologists are pulled in two directions, representing polarized sets of obligations. On the one hand, they have responsibilities as doctors, acting in the best interest of their patients, which ostensibly includes employing the most effective, evidence-based treatment methods. On the other hand, narcologists are prohibited from providing or promoting such methods of treatment and care, such as OST and harm reduction programs, under the threat of criminal and administrative sanctions.

This polarity of obligations creates a hostile working environment, which arguably amounts to violations of narcologists’ right to decent working conditions (Article 7, ICESCR). The CESCR has described the right to work as essential for realizing other human rights and an inherent part of human dignity, and the failure to protect workers from unlawful dismissal (as presumably, contravention of laws prohibiting certain drug dependence treatment methods would lead to) as a state omission that violates the right to work [66]. Moreover, by leaving narcologists with little choice but to uphold repressive methods of working with PWUD and to participate in human rights abuses against PWUD, Russian authorities also prevent narcologists from enjoying productive employment under conditions that safeguard their fundamental political and economic freedoms (Article 6(2), ICESCR), including their rights to due process and freedom of expression.^4^

Some mechanisms in Russia could potentially support narcologists and PWUD to advance human rights and science in drug policy. For example, the Russian Public Mechanism for Monitoring of Drug Policy Reform consists of PWUD and those who support them, including narcologists, lawyers, and journalists. For the last 8 years, the Mechanism has attracted the attention of Russian national authorities as well as UN bodies to Russian drug policy and illuminated the driving force of punitive drug policy behind serious, systematic, and systemic violations of the human rights of PWUD [67]. The Russian Society of Psychiatrists, Russian Society for Evidence-Based Medicine, and other professional organizations of psychiatrists, narcologists, and other medical professionals could jointly petition the Federal Parliament, the Administration of the President, and other federal authorities; pursue strategic litigation; or set up joint working groups to uphold and protect the human rights of PWUD and narcologists. The concept of human rights in patient care could anchor their advocacy.

To date, however, there is only one documented case when a narcologist chose to fight for his right to free expression of his scientific opinion, and indirectly for patients’ right to evidence-based drug dependence treatment by disseminating information about OST on a website [68]. He was prosecuted for distributing drug propaganda and eventually removed the offending materials. The majority of narcologists seemingly have no issue with the status quo. But the declining numbers of narcologists in Russia, in the face of growing demand for drug dependence treatment and care [69], suggests that narcologists opt to leave the profession, rather than openly fight for their rights. As the WHO has noted, the availability of medicine and treatment options can be a powerful source of job satisfaction for health workers [70]. It can also be a strong motivating factor for them to remain in their profession [71]. By imposing unscientific limitations on drug treatment and care, Russian authorities may be dissuading narcologists from their work.

## Conclusions: the way forward

People with drug dependence and narcologists should have shared health objectives, unfettered by scientifically unsound methods of drug dependence treatment and punitive drug policy. In Russia, however, punitive drug policy has severely eroded the relationship between these two groups of potential allies. With the emergence of groups such as the Russian Public Mechanism for Monitoring of Drug Policy Reform, there may be glimmers of hope. Narcologists from this mechanism, for example, have helped document human rights violations against PWUD and participated in meetings with PWUD and advocates about drug policy reform. By working together to uphold their rights, PWUD and narcologists could restore drug users’ trust in narcologists while restoring narcologists’ professional autonomy and independence from law enforcement. On the whole, the Russian public would also benefit from better public health outcomes from drug dependence treatment that is based in science and human rights.

## Abbreviations

CEDAW: Convention on the Elimination of All Forms of Discrimination against Women
CESCR: Committee on Economic, Social and Cultural Rights
DDP: Drug-dependent people
ECHR: European Convention on Human Rights
ICCPR: International Covenant on Civil and Political Rights
ICESCR: International Covenant on Economic, Social and Cultural Rights
OST: Opioid substitution therapy
PWUD: People who use drugs
WHO: World Health Organization

## Footnotes

1 For example, one person remained on medical files 13 years after his last visit to a drug treatment clinic. As a result, he was considered drug dependent and ineligible to drive a vehicle.

2 Previous Law, effective from 1993 to 2012, did not have such an obligation, which originated in Soviet laws.

3 Kurmanayevskiy and others v. Russia (application nos. 62964/10, 58502/11, 55683/13), communicated in May 2014 (three applications concern violations of the right to respect for private life and the right to be free from discrimination by Russian authorities’ refusals to provide applicants with access to OST for drug dependence treatment); Anoshkin v. Russia (application no. 25721/13), not communicated as of July 2017 (the applicant was subjected to torture when police used pain and sufferings due to opioid withdrawal syndrome to coerce his info confession); Ismailova v. Russia (application no. 68868/14), communicated in September 2016 (the case concerns the termination of parental rights of a woman who used drugs).

4 Russia ratified this Convention in 1967. See ILO page Ratifications of C122—Employment Policy Convention, 1964 (No. 122). According to the ILO Employment Policy Convention, 1964 (No. 122), Russia must promote productive employment and ensure that there is the fullest possible opportunity for each worker to qualify for, and to use his/her skills and endowments in a job for which he/she is well suited.

## References

[1] United Nations. The Single Convention on Narcotic Drugs, 1961. 1961. Treaty Series Vol. 976, No. 14152.

[2] President of the Russian Federation. Adopted by the Decree of the President of the Russian Federation No. 690. 2010. Para 23, 48.

[3] Russian Public Opinion Research Center (2017). Press Release No. 3404.

[4] Federal Penitentiary Service of the Russian Federation. Official statistics. 2017.

[5] Golichenko M, Sarang A (2013). Atmospheric pressure: Russian drug policy as a driver for violations of the UN convention against torture and the International Covenant on economic, social and cultural rights. Health Hum Rights..

[6] Human Rights Watch (2007). Rehabilitation required. Russia’s human rights obligation to provide evidence-based drug dependence treatment.

[7] Human Rights Watch (2004). Lessons not learned: human rights abuses and HIV/AIDS in the Russian Federation.

[8] Open Society Institute. The effect of drug user registration laws on people’s rights and health: key findings from Russia, Georgia, and Ukraine. New York: Open Society Institute; 2009.

[9] Levinson L, Torban M (2009). Drug user registration: to follow the law or to follow instructions? Problems of drug user registration in today’s Russia.

[10] Cohen J, Ezer T (2013). Human rights in patient care: a theoretical and practical framework. Health Hum Rights.

[11] UN General Assembly (1966). International covenant on civil and political rights. Resolution.

[12] Council of Europe, European Convention for the protection of human rights and fundamental freedoms, as amended by Protocols Nos. 11 and 14, 4 1950, ETS 5.

[13] UN General Assembly (1966). International covenant on economic, social and cultural rights. Resolution.

[14] General Assembly UN (1979). Convention on the elimination of all forms of discrimination against women. Resolution 34/180.

[15] Beletsky L, Ezer T, Overall J, Byrne I, Cohen J. Advancing human rights in patient care: the law in seven transitional countries. Open Society Foundations. 2013. https://www.opensocietyfoundations.org/sites/default/files/Advancing-Human-Rights-in-Patient-Care-20130516.pdf. Accessed 22 Sep 2017.

[16] Utyasheva L, Elliott R (2008). Effects of UN and Russian influence on drug policy in Central Asia.

[17] Pelipas V. In: Ivanets NI, editor. Ethical aspects of narcology. Guidelines on narcology. Moscow: MIA (Medical Information Agency); 2008. p. 920–9.

[18] Russian Federation (2011). Federal Law No 323-FZ. On fundamentals of protection of health of citizens of the Russian Federation.

[19] Russian Federation (1992). Law N 3185-1. Оn psychiatric care and guarantees of citizens’ rights during its provision.

[20] First All-Russia Congress of Doctors (2012). Code of professional ethics of doctors in the Russian Federation.

[21] Plenary of the Board of the Russian Psychiatrists Association (1994). Code of professional ethics of psychiatrist.

[22] World Health Organization (2009). Guidelines for the psychosocially assisted pharmacological treatment of opioid dependence.

[23] Elovich R, Drucker E (2008). On drug treatment and social control: Russian narcology’s great leap backwards. Harm Reduct J..

[24] Mendelevich VD (2011). Bioethical differences between drug addiction treatment professionals inside and outside the Russian Federation. Harm Reduct J..

[25] Krupitsky E. Short-term intensive psychotherapeutic intervention in narcologi from the prospective of evidence--based medicine. Neurology Messenger Journal. 2010;XLII(3):25-27.

[26] Kurskaya A. Social pressure against drug dependence. Moscow: RIA Novosti; 16; 2011.

[27] USSR. The Conclusion of the Committee of Constitutional Supervision of the USSR No. 8 (2-10). 1990.

[28] Russian Federation (2013). Federal Law No. 313-FZ.

[29] Russian Federation (2013). Federal Law No. 317-FZ.

[30] Russian Federation (2014). Government Decree No. 484.

[31] Russian Federation. Note regarding Bill No. 254761-6. http://asozd2.duma.gov.ru/main.nsf/%28SpravkaNew%29?OpenAgent&RN=254761-6&02. Accessed 30 Aug 2017.

[32] Judicial Department at the Supreme Court of the Russian Federation. Court statistics data. www.cdep.ru/index.php?id=79&item=3832. Accessed 21 Aug 2017.

[33] RosPravosudie. 62 judgments on cases of violations of Article 6.9.1 of the Code of Administrative Offences. https://rospravosudie.com/vidpr-administrativnoe/act-"уклонение+от+лечения"-q/section-acts. Accessed 7 Jul 2017.

[34] National Research Centre of Addictions (2017). Modern narcology: achievements, challenges, future.

[35] Ivanets N, Borisova E. On the implementation and future of the federal targeted program ‘Complex Measures against Drug Abuse and Drug Trafficking in 2005–2009’. Moscow: The Serbsky National Medical Research Centre of Psychiatry and Drug Dependence Treatment; 2006. p. 595–602.

[36] Russian Federation (1998). Federal Law No. 3-FZ.

[37] Russian Federation (2007). Decree of the President of the Russian Federation No. 1374.

[38] Maskas ML (2005). Trafficking drugs: Afghanistan’s role in Russia’s current drug epidemic. Tulsa J. Comparative Int. Law.

[39] Bruin E. Not to do without coercive hospitalization. Medicine Newspaper. No. 93 of December 2, 2011.

[40] Open Society Institute (2009). The effects of drug user registration laws on people’s rights and health.

[41] Ministry of Health, Russian Federation (2015). Order of the Ministry of Health of Russia No. 1034н.

[42] Central Court of Novosibirsk. Judgement of case no. а-3025/2016. 2016. http://sudact.ru/regular/doc/0diQ8WnydeWT/. Accessed 22 Aug 2017.

[43] Rudnichny District Court. Judgment of Case No. 2-746-2010г. 2010. https://rospravosudie.com/court-rudnichnyj-rajonnyj-sud-g-prokopevska-kemerovskaya-oblast-s/act-102895575. Accessed 22 Aug 2017.

[44] Bataysky City Court. Judgment of Case No. 2-203/2012. 2012. https://rospravosudie.com/court-leninskij-rajonnyj-sud-g-rostova-na-donu-rostovskaya-oblast-s/act-103211374. Accessed 22 Aug 2017.

[45] Russian Federation (1995). Family code of the Russian Federation. No 223-FZ. Article 69.

[46] Yakutsky City Court (2013). Judgment of Case No. 2-10789/2013.

[47] Levinson L, Torban M. Drug registry: as per the law or as per an instruction? Regulation of registration of PWUD in the Russian Federation. Moscow: Human Rights Institute; 2009. p. 20–1.

[48] Sovietsky District Court of Astrakhan City (2014). Judgment of Case No. 2-3026/2014.

[49] Arkhangesky District Court, Republic of Bashkortostan (2011). Judgment of Case No. 2-80/2011.

[50] Central Court of Togliatti City (2016). Judgment of Case. No. 2a-6500/2016.

[51] Soviet District Court of Kazan City. Judgment of Case No. 2-698/2012. 2016.

[52] Russian Federation (1993). Fundamentals of law of the Russian Federation about health protection of citizens. Law No. 5487-1.

[53] USSR. On the approval of the fundamentals of laws of the Union of SSR and Union’s republics about health protection. Article 4. Law of the USSR. 1969.

[54] Golichenko M (2016). Tuberculosis, stigma and drug control: a case from Russia.

[55] Special Profedures of the UN Human Rights Council. Communication between the Russian Federation and two of the UN Special Rapporteurs. 2013. All Health (2002–7) G/SO 214 (89–15) RUS 5/213. https://spdb.ohchr.org/hrdb/24th/Public_-_AL_Russia_15.07.13_(5.2013)_Pro.pdf. Accessed 27 Aug 2017.

[56] Human Rights Council, United Nations General Assembly. Report of the Special Rapporteur on the right of everyone to the enjoyment of the highest attainable standard of physical and mental health, Anand Grover. Addendum 1. A/HRC/17/25/Add.1. 2011.

[57] Grover A (2010). Report of the Special Rapporteur on the right of everyone to the enjoyment of the highest attainable standard of physical and mental health.

[58] Human Rights Council, United Nations General (2013). Report of the Special Rapporteur on torture and other cruel, inhuman or degrading treatment or punishment, Juan E. Méndez.

[59] Committee on Economic, Social and Cultural Rights (2011). Concluding observations on Russia.

[60] Andrey Rylkov Foundation for Health and Social Justice and the Canadian HIV/AIDS Legal Network (2010). Report to CESCR on Russia.

[61] Human Rights Commission (2015). Concluding observations on Russia.

[62] Committee on the Elimination of All Forms of Discrimination against Women (2015). Concluding observations on Russia.

[63] Russian Federation (2001). Russian Federation Code of Administrative Offences. Article 6.13. Law No. 195-FZ.

[64] Russian Federation (1996). Criminal Code of the Russian Federation. Article 230. Law No 63-FZ.

[65] Zlobin A, Kovalevsky A. Revolucia Doz. Newsweek. 2007.

[66] Committee on Economic, Social and Cultural Rights (2006). General Comment No. 18: The Right to Work (Article 6 of the Covenant).

[67] HIV/AIDS Legal Network. Search results for: Russia. 2017. http://www.aidslaw.ca/site/?s=russia&searchsubmit=Search&lang=en (Accessed 22 Sep 2017).

[68] Partiff T (2006). Vladimir Mendelevich: fighting for drug substitution treatment. Lancet.

[69] Kirzhanova V (2017). Main indicators of the functioning of the narcological service in the Russian Federation in 2015–2016: statistics.

[70] World Health Organization (2006). Treat, train, retain: the AIDS and health workforce plan: report on the consultation on AIDS and human resources for health.

[71] Human Resources for Health. Overcoming the crisis. Joint learning initiative: The President and Fellows of Harvard College. Washington: Communications Development Incorporated in Washington; 2004. p. 79.

